# Treadmill running on neuropathic pain: via modulation of neuroinflammation

**DOI:** 10.3389/fnmol.2024.1345864

**Published:** 2024-06-26

**Authors:** Wei-Chun He, Shuang-Long Hou, Kai-Bin Wang, Ning Xu, Ke Li, Ting Xiong, Jing Luo

**Affiliations:** ^1^Department of Rehabilitation Medicine, General Hospital of NingXia Medical University, Yinchuan, China; ^2^Department of Sport Rehabilitation, Xi’an Physical Education University, Xi’an, China

**Keywords:** treadmill running, neuropathic pain, neuroinflammation, analgesic mechanism, review

## Abstract

Neuropathic pain is a type of chronic pain caused by an injury or somatosensory nervous system disease. Drugs and exercise could effectively relieve neuropathic pain, but no treatment can completely stop neuropathic pain. The integration of exercise into neuropathic pain management has attracted considerable interest in recent years, and treadmill training is the most used among exercise therapies. Neuropathic pain can be effectively treated if its mechanism is clarified. In recent years, the association between neuroinflammation and neuropathic pain has been explored. Neuroinflammation can trigger proinflammatory cytokines, activate microglia, inhibit descending pain modulatory systems, and promote the overexpression of brain-derived neurotrophic factor, which lead to the generation of neuropathic pain and hypersensitivity. Treadmill exercise can alleviate neuropathic pain mainly by regulating neuroinflammation, including inhibiting the activity of pro-inflammatory factors and over activation of microglia in the dorsal horn, regulating the expression of mu opioid receptor expression in the rostral ventromedial medulla and levels of γ-aminobutyric acid to activate the descending pain modulatory system and the overexpression of brain-derived neurotrophic factor. This article reviews and summarizes research on the effect of treadmill exercise on neuropathic pain and its role in the regulation of neuroinflammation to explore its benefits for neuropathic pain treatment.

## 1 Introduction

Pain is caused by the activation of nociceptors, which are activated in three ways: mechanical, chemical, and thermal stimulation. When a tissue is damaged or an inflammatory reaction occurs, the concentrations of chemicals in the tissue increases. When the concentration exceeds the nociception threshold, nociceptors are activated, and then nociceptive information is transmitted to the dorsal root ganglia (DRGs) for initial integration, ultimately reaching the cerebral cortex and causing pain. When the concentrations of chemicals causing pain decrease, pain gradually decreases until it disappears (McHugh and McHugh, 2000). Neuropathic pain refers to pain caused directly by lesions or diseases affecting the somatosensory nervous system and is typically characterized by spontaneous ongoing or shooting pain (Baron et al., 2010). Bibliometric analysis shows that in the past 20 years, research on neuropathic pain has expanded, emerging as a well-developed and prospective field of medical study (Xu et al., 2022). Neuropathic pain can lead to dysfunction and disability, reduce the quality of life of patients, and imposes considerable economic burden on society (Ruiz-Negrón et al., 2019). The incidence of neuropathic pain is relatively high worldwide. A systematic review of epidemiological studies has shown that the prevalence of neuropathic pain is 6.9–10% (van Hecke et al., 2014). In patients with multiple sclerosis, the incidence rate of neuropathic pain is even as high as 26.8% (Rodrigues et al., 2023).

The treatment of neuropathic pain is a long-term and complex process that requires the full consideration of a patient’s treatment effectiveness and cost-effectiveness (Critchlow et al., 2017). The clinical treatment of neuropathic pain usually includes drug therapy and nondrug strategies. Currently, evidence-based medicine supports the use of tricyclic antidepressants, serotonin noradrenaline reuptake inhibitors, pregabalin, and gabapentin as first-line treatments for neuropathic pain (Finnerup et al., 2015; Moisset et al., 2020). However, insufficient response to drug therapy prevents most patients with neuropathic pain from achieving the expected benefits of drug therapy. Less than 50% of patients with neuropathic pain are effectively treated with medication (Finnerup et al., 2016; Serrano Afonso et al., 2021). Medication treatment for neuropathic pain cannot achieve expected results and is related to a decrease in drug efficacy and affected by adverse drug reactions (Serrano Afonso et al., 2021). Considering intervention effectiveness and cost-effectiveness, researchers have turned their attention to nondrug treatment, which mainly includes exercise therapy, which is an effective treatment for neuropathic pain (Zhang Y. H. et al., 2021; Tatikola et al., 2022). Exercise can relieve pain effectively, and aerobic exercise produces an overall analgesic effect on proximal and distal motor sites (Wewege and Jones, 2021). In recent years, exercise therapy has drawn the attention of researchers. Treadmill exercise is preferred over other exercise therapies (such as swimming, cycling, and jumping rope). It is easy to perform and safe for patients, requires a small space, and involves the multiple aspects of exercise: joint range of motion, muscle strength, and balance ability. Relieving patients’ pain and causing almost no side effects, running on a treadmill not only alleviates neuropathic pain and fatigue but also effectively improves gait, balance, flexibility, coordination, and quality of life (Field-Fote et al., 2005; Chang et al., 2021; Jung et al., 2021). In addition, treadmill exercise can regulate the levels of inflammatory cytokines and pain-related signal transduction, thereby exerting specific analgesic effects (Hutchinson et al., 2004; Cobianchi et al., 2010; López-Álvarez et al., 2015; Nees et al., 2016; Sumizono et al., 2018; Tashiro et al., 2018). From the perspective of the pathological mechanism of neuropathic pain induced by neuropathic inflammation, the occurrence of neuroinflammation is closely related to inflammatory cytokines, which are important factors in inflammatory diseases and key mediators in neuropathic pain (Sommer et al., 2018). Exercise therapy may relieve neuropathic pain and other types of pain by regulating inflammatory cytokines (Chen et al., 2012; Yoon et al., 2015; Sluka et al., 2018; Chhaya et al., 2019; Dugan et al., 2020; Wang et al., 2024). Therefore, in recent years, the mechanism of pain reduction by treadmill exercise has been increasingly explored from the perspective of inflammatory factors. This review is based on research on the relationship between neuropathic inflammation and neuropathic pain and describes the pathologic mechanism by which exercise relieves nerve pain and regulate neuropathic inflammation.

## 2 Neuroinflammation and neuropathic pain

Tissue damage induces inflammation, in which immune cells, other cells, and body fluids produce inflammatory mediators, namely, nerve growth factor, NLRP3-related inflammasome, and toll-like receptor 4, which can cause pain hypersensitivity (Oh et al., 2001; Nicol and Vasko, 2007; Bobinski et al., 2018; Sommer et al., 2018; Sun et al., 2021; Szabo-Pardi et al., 2021). Several possible pathways may mediate neurogenic pain caused by inflammatory mediators.

### 2.1 Pro-inflammatory cytokines and excessive activation of microglia

Microglia play an important role in neuropathic pain and neuroinflammation. Tissue damage causes local inflammation, releasing mediators, such as chemokines and neurotrophic factors. These substances can activate the microglia, leading to the onset of neuropathic pain. Subsequently, the microglia recruit astrocytes and release inflammatory factors, strengthening and spreading neuropathic pain (Wieseler-Frank et al., 2005; Vallejo et al., 2010). Interleukin 1β (IL-1β) is a key mediator of the interaction between neuropathic pain and neuroinflammation. After a central nervous system injury, activated glial cells can release the inflammatory mediator IL-1β in the central nervous system, driving the occurrence and enhancement of neuropathic pain (del Rey et al., 2012). Shao et al. established a neuropathic pain model by disrupting the ventrolateral orbital cortex (VLO) of rats. The expression level of IL-1β in the contralateral VLO of rats considerably increased, indicating that the pro-inflammatory cytokine IL-1β mediates neuropathic pain after a central nervous system injury (Shao et al., 2015). Therefore, regulating excessive IL-1β in the spinal cord and brain regions after nerve injury may relieve neuropathic pain. Amyloid β (Aβ) is surrounded by many inflammatory cytokines, reactive astrocytes, and microglia, forming Aβ plaques. On the one hand, the activated microglia and inflammatory mediators in the pathological state promote the production and accumulation of Aβ. On the other hand, Aβ itself can induce neuroinflammation and excessive microglial activation. Therefore, Aβ, microglia, and neuroinflammation form a vicious cycle, jointly leading to the generation and intensification of neuropathic pain (Wyss-Coray et al., 2003; Zaheer et al., 2008). Targeted intervention with Aβ can effectively reverse this vicious cycle. When pathological inflammation occurs in recipient tissues, cells, such as glial cells, produce nitric oxide (NO) in response to NO synthase expression (Coleman, 2001). The production of NO in turn aggravates the inflammatory response and forms a cycle, which causes excitotoxic cell death and neuronal damage, thus leading to neuropathic pain (Olivera et al., 2016). Therefore, treating neuropathic pain diseases with NO is reasonable (Liy et al., 2021).

### 2.2 Inhibition of the pain modulatory system

Tissue damage causes neuroinflammation, in which nociceptors are activated, and nociceptive information is transmitted to DRGs for initial integration, ultimately reaching the cerebral cortex, and pain occurs. This process is regulated by the descending pain modulatory system, which originates in the brainstem and terminates in the spinal dorsal horn. The rostral ventromedial medulla (RVM) and midbrain periaqueductal gray (PAG) are the important parts of the inhibitory system, and the RVM receives information projection from the PAG (Pertovaara and Almeida, 2006). Some scholars believe that descending pain inhibition systems can be either inhibitory or promoting (Porreca et al., 2002; Vanegas, 2004). Preliminary evidence provided by animal models suggests that the descending pain inhibition system primarily inhibits primary hyperalgesia, promotes secondary pain (Vanegas, 2004). Carlson et al. (2007) studied the RVM neurons in spinal nerve ligation rats and observed changes in on-cells and off-cells responses to mechanical stimuli in the early stages of nerve injury. Specifically, the increased mechanical sensitivity in both on-cells and off-cells of RVM indicates an enhanced specific response of pain regulating neurons in RVM after nerve injury, ultimately leading to abnormal neuropathic pain and hyperalgesia. Endogenous opioid drugs, such as met-enkephalin and leu-enkephalin, are widely distributed in the PAG and RVM, and γ-aminobutyric acid (GABA) is a key neurotransmitter for the projection of PAG–RVM neurons. When harmful stimuli occur, the body releases endogenous opioid substances, which act on the mu opioid receptor (MOR) in the GABA inhibitory neuron, relieving pain (Bagley and Ingram, 2020). Neuroinflammation can cause damage to the descending pain regulation system. The hypersensitivity of neurons and glial activation after spinal cord injury disrupts the balance of chloride ions, glutamate, and GABA distribution in the spinal dorsal horn and results in chronic neuropathic pain (Gwak and Hulsebosch, 2011). A rat model of Parkinson’s disease (PD) showed a substantial decrease in the withdrawal threshold for nociceptive stimuli, accompanied by an increase in pro-inflammatory cytokines, such as IL-1β, interleukin-6 (IL-6), and tumor necrosis factor α (TNF-α) receptors, and a decrease in GABA level (Zhuang et al., 2016). Exogenous opioid drugs can activate descending pain inhibition system by relieving the intrinsic GABAergic inhibition of PAG neurons, which project to the RVM (Winters et al., 2022). After the PIC receptor in the GABA concentration was blocked, the withdrawal threshold in the PAG of PD rats markedly improved (Zhuang et al., 2016). Meanwhile, the balance of potassium chloride cotransporter-2 (KCC2) and Na + − K + -2Cl^−^cotransporter 1 (NKCC1) are crucial for determining the strength and polarity of GABA (Yousuf and Kerr, 2016). After nerve injury, an imbalance between KCC2 and NKCC1 (manifested by significant upregulation of NKCC1 and synchronous downregulation of KCC2) can lead to a transition of GABA neurotransmission from an inhibitory state to an excited state, promoting the occurrence of neuropathic pain (Hasbargen et al., 2010). Therefore, targeting the PAG–RVM circuitry and GABA to regulate the pain modulatory system seems possible relieve the neuropathic pain.

### 2.3 Regulation of BDNF levels

Brain-derived neurotrophic factor (BDNF) is synthesized by small neuronal cell bodies in DRGs. BDNFs are associated with the regulation of nociceptive signaling during neuropathic pain (Obata and Noguchi, 2006). Tyrosine receptor kinase B (TrkB) is a high-affinity receptor of BDNF. The BNDFs in the spinal cord of rats with brachial plexus avulsion greatly increased after its connection to the TrkB receptor, participating in nociceptive neural regulation. The intrathecal administration of the TrkB-specific antibody K-252, a mechanical hyperalgesia in model rats, while reducing the expression levels of BDNF and TrkB, indicating that the BDNF–TrkB pathway is involved in neuropathic pain caused by peripheral nerve injury (Zhao et al., 2022). Similarly, in CCI sciatic nerve model mice, CCI led to the overexpression of the BDNF protein in the midbrain limbic reward system, thereby mediating the regulation of neuropathic pain (Zhang et al., 2017). Meanwhile, BDNFs participate in the sympathetic nerve germination of the dorsal root ganglion in PNI rats, leading to pain sensitization after injury (Deng et al., 2000). In addition, BDNFs can lead to neuropathic pain by activating the microglia in the spinal dorsal horn, and injecting BDNF scavenger into its sheath can effectively reduce the behavioral signs of neuropathic pain in spared nerve injury (SNI) model rats (Zhou et al., 2011). Therefore, the overexpression of BDNF in the spinal dorsal horn, DRGs, and central nervous system can lead to neuropathic pain, and targeted intervention with BDNF is a promising treatment strategy for neuropathic pain treatment.

## 3 Therapeutic mechanisms of exercise on neuropathic pain

**Figure 1:**
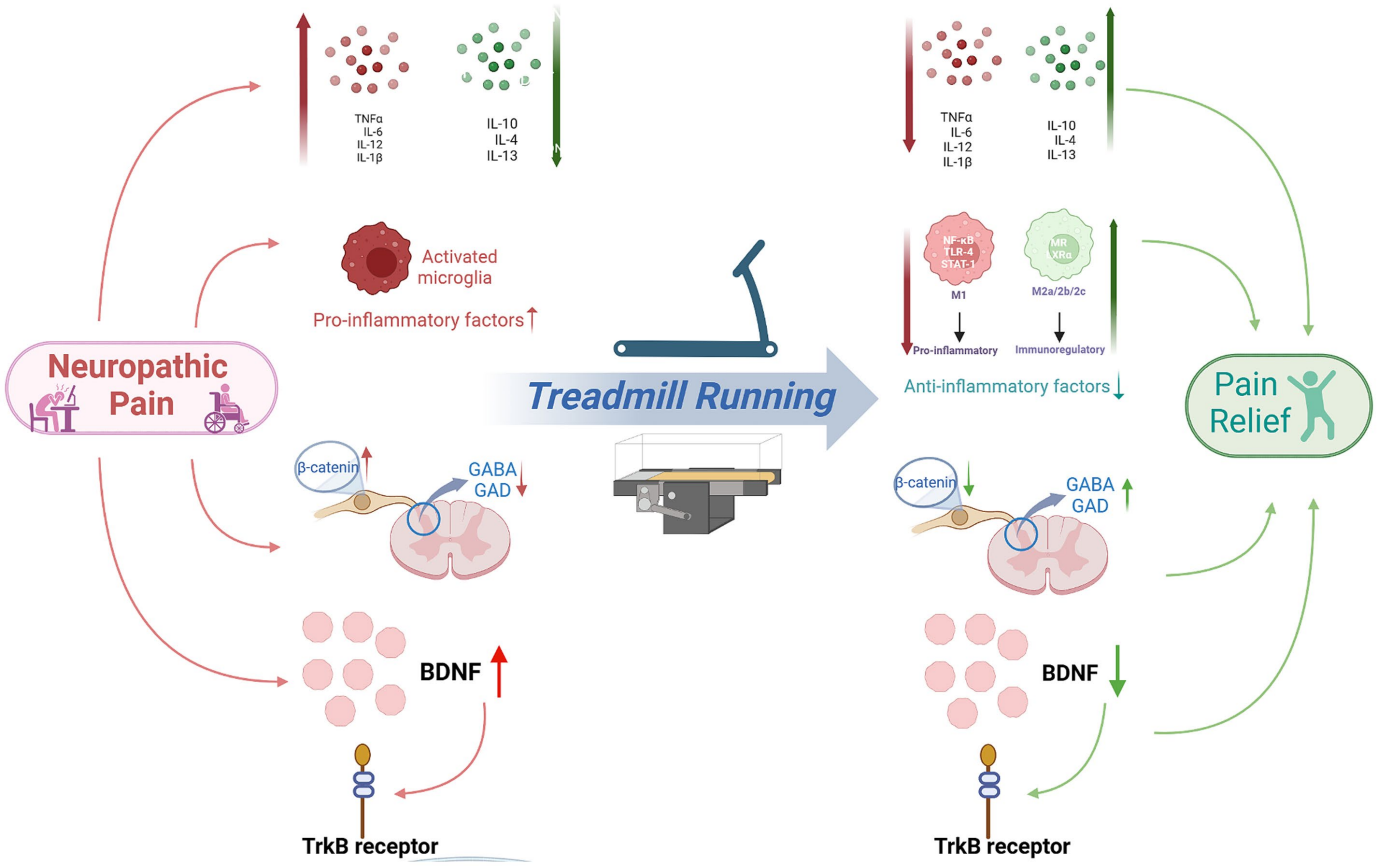
Treadmill running alleviates neuropathic pain by inhibiting neuroinflammation. The mechanisms involved include the regulation of inflammatory cytokines and glial cells, the activation of the pain inhibitory system, synaptic plasticity and neuronal regulation, etc. IL-6, interleukin-6; IL-10, Interleukin-10; M2, macrophages2; BDNF, brain-derived neurotrophic factor; NGF, nerve growth factor; IL-4, interleukin-4; M1, macrophages1; GABA, gamma-aminobutyric acid; GAD, glutamic acid decarboxylase; TNFα, tumor necrosis factor-α.

When inflammation is induced by nervous system injury, changes occur in the spinal cord and brain regions involved in pain processing (Costigan et al., 2009), and patients with pain due to neuroinflammation usually present with persistent discomfort or blunt pain. The occurrence of inflammation can activate the microglia in the spinal cord and cause them to release inflammatory cytokines, which stimulate nociceptors and produce neuropathic pain (Vallejo et al., 2010; Vanelderen et al., 2010). Nerve injury may lead to the decrease in GABA concentration and the change in GABA polarity, so there lead to disruption of the inhibitory of descending pain modulatory system, exacerbating neuropathic pain (Porreca et al., 2002; Vanegas, 2004; Pertovaara and Almeida, 2006; Hasbargen et al., 2010; Gwak and Hulsebosch, 2011; Zhuang et al., 2016). In addition, inflammation can regulate the levels of BDNFs, leading to nociceptive hypersensitivity reactions, which induce neuropathic pain (Sikandar et al., 2018). Exercise can reduce pain sensitivity and increase pain threshold in patients (Belavy et al., 2021; Zhang Y. H. et al., 2021; Tatikola et al., 2022). This article summarizes research related to the regulation of neuropathic pain by treadmill running through neuroinflammatory mechanisms (Table 1). Treadmill running can inhibit the activity of pro-inflammatory cytokines and excessive activation of microglia (Tamakoshi et al., 2022), activate the descending pain modulatory system (Kami et al., 2016), and regulate the levels of BDNF (Sumizono et al., 2018), thereby relieving neuropathic pain (Figure 1).

**Table 1:** General features of studies on the relief of neuropathic pain by inhibition of neuroinflammation by treadmill running.

Reference	Model	Exercise (treadmill)	Related cytokines/cells/proteins	Functions	Involved in pathways/channel	Change
Chen et al. (2012)	CCI Rats	1.8 km/h, 3 days/week, 6 weeks	TNF-α, IL-1β	Pro-inflammatory cytokines	-	↓
Supruniuk et al. (2020)	Wistar rats	18 m/min, 30 min/day	NO	Pro-inflammatory cytokines	-	↓
Cobianchi et al. (2010)	CCI Mice	20 cm/s, 1 h/day, 5 days/week, 2 weeks	Microglia, astrocytes	Pro-inflammatory	-	↓
Mu et al. (2022)	AD Mice	18 m/s, 30 min/days, 3 days/week, 12 weeks	Aβ, GSK3β, IL-1β, IL-6,TNF-α	Pro-inflammatory proteins/kinase/cytokines	-	↓
Binda et al. (2020)	PD Rats	10 m/s, 40 min/day, 3 days/week, 4 weeks	MOR	Opioid receptor	-	↑
Kami et al. (2016)	PSL Mice	7 m/min, Week 1: 10 min/day, 5 days/week Week 2: 20-60 min/day (increase by 10 min/day), 3 weeks	GABA, GAD65/67	Proteins		↑
Li et al. (2020)	SCI Rats	6 m/min, 2times/day, 5 days/weeks, 4 weeks	GAD65/67	Proteins	TrkB	↑
Cho et al. (2021)	PSL Mice	8 m/min, everyday	GABA, GAD 65/67	Proteins	Wnt/β-catenin	↑
Bobinski et al. (2018)	PNI Mice	10 m/min, 30 min/day, 5 days/week, 2 weeks	BDNF, β-NGF, IL-4, M1	Pro-inflammatory cytokines, macrophages	GFAP and Iba1	↓
Bai et al. (2022)	PNI Mice	7 m/min, Week 1: 10 min/day, 5 days/week Week 2: 20-60 min/day (increase by 10 min), 5 days/week	BDNF, M1	Pro-inflammatory cytokines, macrophages	BDNF/AKT/mTOR	↓
Lu et al. (2021)	Stroke Rats	12 m/min， 30 min/day, 3 days/week, 1 week	IL-4, IL-1ra, IL-5, M2	Anti-inflammatory cytokines		↑

CCI, chronic constriction injury; PNI, peripheral nerve injury; SCI, spinal cord injury; PSL, partial sciatic nerve ligation; AD, Alzheimer’s; PD, Parkinson’s disease; IL-6, interleukin-6; IL-10, Interleukin-10; M2, macrophages2; BDNF, brain-derived neurotrophic factor; NGF, nerve growth factor; IL-4, interleukin-4; M1, macrophages1; GABA, gamma-aminobutyric acid; GAD, glutamic acid decarboxylase; TNFα, tumor necrosis factor-α; NO, nitric oxide; MOR, mu Opioid receptor; Aβ, Amyloid beta.

### 3.1 Regulation of inflammatory cytokines and glial cells

Microglia are immune cells in the central nervous system. The angiogenesis of the nervous system, induction of apoptosis, phagocytosis, removal of dead cells, and development of protruding remodeling are all affected by the microglia (Eyo and Dailey, 2013). Notably, the generation of neuropathic pain is closely related to the activation of microglia (Wyss-Coray et al., 2003; Zaheer et al., 2008; Vallejo et al., 2010; Liu et al., 2020). Exercise can reduce neuropathic pain by regulating the levels of pro-inflammatory cytokines and play a specific analgesic role. The overproduction of TNF-α and IL-1β in CCI sciatic nerve mice considerably decreased after treadmill exercise (Chen et al., 2012). A PNI mouse model was established by compression injury of the sciatic nerve (Bobinski et al., 2018). After two weeks of treadmill training for 30 min (10 m/min, 5 days a week), the mice were tested for the expression of anti-inflammatory factors. The percentage of M2 macrophages (secreting anti-inflammatory cytokines) increased after treadmill training. Meanwhile, Lu et al. found that treadmill training increased the expression of interleukin-4 (IL-4), increased the inhibitory effect of anti-inflammatory M2 cells on M1 microglia and the promotion effect on M2 microglia, and reduced the markers of pro-inflammatory M1 cells in microglia, thereby inhibiting an inflammatory response. The percentage of anti-inflammatory cytokines (IL-4, IL-1ra, and IL-5) returned to pre-injury levels, and the percentage of M1 macrophages (secreting proinflammatory cytokines) decreased (Lu et al., 2021). After treadmill exercise at different speeds, the level of NO in male rats markedly decreased, and this benign change may have alleviated NO-mediated neuropathic pain (Supruniuk et al., 2020). Aβ can induce neuroinflammation and neuropathic pain (Wyss-Coray et al., 2003; Zaheer et al., 2008). Loreto et al. found that treadmill exercise can activate the anti-amyloidogenic production pathway in mice, reducing the accumulation of Aβ (Di Loreto et al., 2014). After 12 weeks of treadmill training, AD model mice showed considerable decrease in the levels of oligomers (Aβ dimers and trimers) and pro-inflammatory cytokines (including IL-1β, IL-6, and TNF-α) (Mu et al., 2022). Given the pathological contribution of amyloid protein to neuropathic pain, this change may reduce the activation of microglia and the release of pro-inflammatory factors and can thereby alleviate neuropathic pain. In addition, the expression levels of microglia and astrocytes in CCI sciatic nerve mice decreased after short-term treadmill exercise, which may have alleviated neuropathic pain by improving the excessive activation of microglia during neuroinflammation (Cobianchi et al., 2010). Furthermore, treadmill running can inhibit the excessive activation of astrocytes and microglia by inhibiting the activity of GSK3β kinase, thereby reducing the production of pro-inflammatory cytokines and relieving neuropathic pain (Mu et al., 2022). Overall, we can conclude that treadmill training can relieve neuropathic pain by regulating the levels of inflammatory cytokines and the activation of glial cells.

### 3.2 Activation of the pain modulatory systems

The PAG and RVM are the key components of the descending pain inhibition system and pathological contributors to pain hypersensitivity (Porreca et al., 2002; Vanegas, 2004; Zhuang et al., 2016) In a mouse model of chronic musculoskeletal injury, wheel running greatly reduced pain hypersensitivity. However, when the regulation of the PAG and RVM was blocked with naloxone, the pain relief after exercise was reversed (Brito et al., 2017). This result indicated that the PAG and RVM are involved in the mediation of the analgesic process of wheel running. MOR is the main opioid receptor that mediates pain relief and is widely expressed in PAG and RVM neurons (Matthes et al., 1996; Bagley and Ingram, 2020). Binda et al. established a neuropathic pain model of PD mice and administered exercise intervention three times a week (40 min each time). After the training, the pain was alleviated, and the expression of MOR in the PAG in the thalamus was greatly enhanced. Therefore, treadmill exercise can regulate the MOR in the PAG, inhibiting pain (Binda et al., 2020). GABA is mainly synthesized through the deacidification of glutamate, mediated by glutamate decarboxylase 65 (GAD65) and glutamate decarboxylase 67 (GAD67). After nerve injury, the decrease in GABA concentration and GAD expression leads to a decrease in GABA inhibitory function, thereby increasing neuropathic pain (Gwak and Hulsebosch, 2011; Zhuang et al., 2016; Li et al., 2019). Spinal cord stimulation in PSL model rats increased GABA and GAD65/67 levels, which in turn improved neuropathic pain behavior in rats. Treadmill training can maintain GABA retention in interneurons and nerve terminals and maintain GAD65/67 levels, thereby relieving neuropathic pain (Kami et al., 2016). Dugan et al. conducted a two-year treadmill training test on a rat model of SCI and found that intensive exercise training relieve neuropathic pain by reducing the inflammatory process of the spinal cord and promoting the recovery of spinal cord depression (Dugan et al., 2021). Exercise training can also modulate GAD65/67 expression through TrkB signaling and ameliorate neuropathic pain in rats with SCI (Li et al., 2020). Moreover, treadmill exercise increases the level of GAD65/67 while increasing the expression of KCC2 in SCI model mice, reversing the excitability of spinal dorsal horn neurons, thereby alleviating neuropathic pain (Dugan et al., 2020; Li et al., 2022). A similar study found that high-intensity treadmill exercise significantly reduced early pain hypersensitivity in sciatic nerve injury, and this mechanism may stem from blocking early pain sensitization mechanisms, such as NKCC1/KCC2 disregulation, and this will cause GABA neurotransmission to revert from excitability to inhibition (López-Álvarez et al., 2015).

The classical Wnt pathway is an indicator of pain, which increases inflammatory cytokines in the DRG and spinal dorsal horn (Zhang Y. et al., 2021). Wnt binds to its receptor Frizzled (Fzd), and Disheveled recruited by Fzd leads to the phosphorylation of RP5/6. Phosphorylated RP5/6 recruits axin onto the membrane, disrupting the decomposition of the complex and resulting in the accumulation of β-catenin, which is then translocated from the cytoplasm to the nucleus to activate T lymphocyte cytokines or lymphenhancer series transcription factors; these processes aggravate the mechanical pain hypersensitivity induced by SNI (Wisniewska et al., 2010; Silva-García et al., 2019). Treadmill training could regulate neuropathic pain after PNI by delaying the Wnt/β-catenin signaling pathway in DRG neurons (Cho et al., 2021). These results indicated that the regulation of endogenous opioid drugs by the RVM and PAG, the maintenance of GABA and GAD levels, and the delay of Wnt/β-catenin signaling pathway can lead to the activation of the descending pain inhibitory system and then exert a suppressive effect on neuropathic pain. These factors can be induced through exercise.

### 3.3 Regulation of BDNF

The overexpression of BDNF during tissue injury and neuroinflammation is a pathological contributor to neuropathic pain (Deng et al., 2000; Zhang et al., 2017; Zhao et al., 2022), and treadmill exercise can reverse this process. Sumizono et al. established a CCI sciatic nerve model. The model mice underwent five weeks of treadmill exercise. After training, the neuropathic pain of the model mice was alleviated through the regulation of glial cell activation and BDNF expression in the spinal dorsal horn (Sumizono et al., 2018). Similarly, the proliferation of microglia in the spinal dorsal horn and overexpression of BDNF in the microglia of the SNI rats considerably improved after the treadmill exercise (López-Álvarez et al., 2015; Bobinski et al., 2018). Exercise training may regulate the microglial polarization through a BDNF/AKT/mTOR signaling–mediated autophagy flux, thereby alleviating neuroinflammation (Bai et al., 2022). Therefore, treadmill exercise can reverse the overexpression of BDNF during tissue damage and neuroinflammation, thereby alleviating neuropathic pain.

## 4 Conclusion and future direction

Most patients suffer from neuropathic pain, and no complete clinical treatment is available yet. Therefore, pain management for patients with neuropathic pain is urgently needed. Through literature search, we found that exercise therapy exerts an effect that relieves neuropathic pain. Treadmill running has been preferred for the treatment of neuropathic pain because of its simple operation and good therapeutic effect. This article reviews the mechanism and role of treadmill running in neuropathic pain relief. In summary, treadmill exercise can regulate inflammatory factors and overactivated microglia, induce the descending system to inhibit pain, regulate BDNF level, and ultimately alleviate neuroinflammation and neuropathic pain. This article provides a basis for the application of treadmill running in neuropathic pain. However, not all intensity exercise programs are beneficial for neuropathic pain. Therefore, the sustained effects and optimal exercise dose of treadmill running on neuropathic pain relief must be further explored.

## Author contributions

W-CH: Writing – original draft, Writing – review & editing. S-LH: Writing - review & editing. K-BW: Writing – original draft, Writing – review & editing. NX: Writing – review & editing. KL: Writing – original draft. TX: Writing – original draft. JL: Writing - review & editing.
